# Constraints on global temperature target overshoot

**DOI:** 10.1038/s41598-017-14503-9

**Published:** 2017-11-07

**Authors:** K. L. Ricke, R. J. Millar, D. G. MacMartin

**Affiliations:** 1 Scripps Institution of Oceanography, UC San Diego, La Jolla, USA; 2 School of Global Policy and Strategy, University of California, San Diego, La Jolla, USA; 3 Environmental Change Institute, University of Oxford, Oxford OX1 3QY, UK; 4 Mechanical and Aerospace Engineering, Cornell University, Ithaca, NY 14850 USA

## Abstract

In the aftermath of the Paris Agreement, the climate science and policy communities are beginning to assess the feasibility and potential benefits of limiting global warming to 1.5 °C or 2 °C above preindustrial. Understanding the dependence of the magnitude and duration of possible temporary exceedance (i.e., “overshoot”) of temperature targets on sustainable energy decarbonization futures and carbon dioxide (CO_2_) removal rates will be an important contribution to this policy discussion. Drawing upon results from the mitigation literature and the IPCC Working Group 3 (WG3) scenario database, we examine the global mean temperature implications of differing, independent pathways for the decarbonization of global energy supply and the implementation of negative emissions technologies. We find that within the scope of scenarios broadly-consistent with the WG3 database, the magnitude of temperature overshoot is more sensitive to the rate of decarbonization. However, limiting the duration of overshoot to less than two centuries requires ambitious deployment of both decarbonization and negative emissions technology. The dependencies of temperature target overshoot’s properties upon currently untested negative emissions technologies suggests that it will be important to consider how climate impacts depend on both the magnitude and duration of overshoot, not just long term residual warming.

## Introduction

Two primary technology types are implemented in contemporary economic models of climate change mitigation to limit climate system warming from anthropogenic gases. First, there are technologies that reduce CO_2_ and other greenhouse gas emissions from the energy supply by replacing carbon-emitting fuels with low or no-carbon alternatives (referred to as *decarbonization* technologies hereafter). Second, there are technologies that capture and sequester CO_2_ already in the atmosphere (*negative emissions* technologies hereafter).

There exists a large portfolio of decarbonization technologies, including renewable energy generation (such as wind and solar power), nuclear power and fossil fuel power with carbon capture and storage. While there are engineering and cost-effectiveness challenges associated with decarbonization technologies, there is also a long history of development and significant present-day deployment of some decarbonizing technologies. Pathways for implementation have been extensively explored in the academic literature and policy arena^1^.

Negative emissions technologies are associated with a distinct set of uncertainties, including remaining largely untested at scale^2^. Integrated assessments of climate policy typically only consider bioenergy with carbon capture and storage (BECCS) – which is both a decarbonization and negative emissions technology— and land use change (e.g., reforestation) as possible negative emissions sources^3,4^. While there are proposed alternative approaches for capturing CO_2_ from the air (for example, direct air capture or enhanced weathering), all are associated with comparatively large engineering and economic uncertainties^5^. In the case of BECCS and other biological or biogeochemical approaches to capturing carbon, there exist uncertain, but hard, biophysical limits on the maximum deployment of negative emissions such as land and water availability^6^, as well as considerable uncertainty on future policy commitment to development and deployment^7^.

In December 2015, the global community affirmed a goal of ‘holding the increase in global average temperature to well below 2 °C above pre-industrial levels and to pursue efforts to limit temperature increases to 1.5 °C’^8^. Limiting warming to 1.5 degrees in 2100 will require very rapid transformation of the energy system over the rest of the century^9^ and even many mitigation scenarios that aim to restrict temperatures to 2 °C or less require substantial deployments of negative emissions technologies^2^. However, it is currently unknown whether decarbonization and negative emissions technologies can be deployed quickly enough, and on sufficient scale, to avoid exceeding 1.5 °C peak warming. Thus an “overshoot” of 1.5 °C – that is, a period of time in which the global temperature increase over pre-industrial exceeds a 1.5 °C warming target—appears likely even under such ambitious mitigation scenarios.

Recent research has been undertaken to characterize the challenges associated with closing the mitigation gap between business as usual, the Paris emissions pledges and various temperature stabilization scenarios^10–13^. The Intergovernmental Panel on Climate Change (IPCC) Working Group 3 (WG3) scenario database^3^ is often used to provide a representative range of potential future emissions time series in these analyses^2,14^. While the importance of negative emissions in overshoot scenarios is widely recognized^15^, the respective contributions of decarbonization and negative emissions in these scenarios are not explicit. This is due in part to the fact that BECCS – the only significant long-term negative emissions technology deployed in these scenarios – both generates energy that can displace fossil fuels (i.e., decarbonization) and sequesters atmospheric CO_2_ (i.e., negative emissions). Given the distinct uncertainties associated with deployment of negative emissions technologies than decarbonization, this lack of technological attribution obscures straightforward assessment of the constraints upon meeting specific temperature targets under specific timelines and conditions (including overshoot).

**Figure 1:**
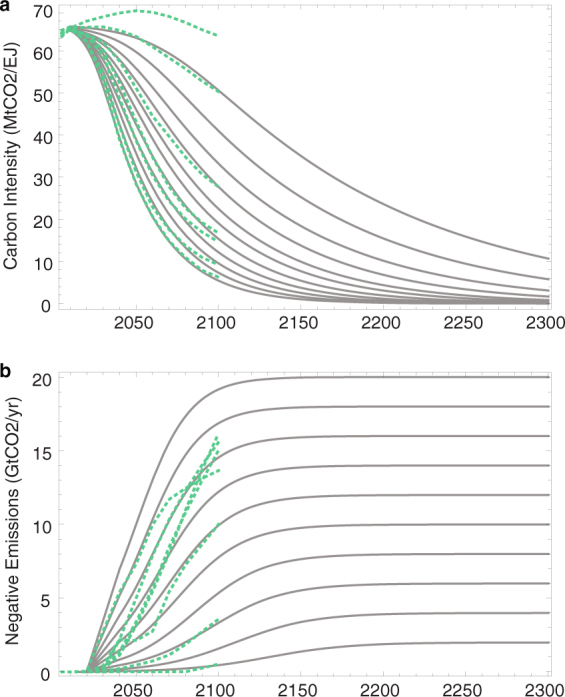
Decarbonization and negative emissions scenarios. Black lines show the scenarios explored in this analysis for (**a**) carbon intensity (MtCO2/EJ), a proxy for decarbonization, and (**b**) negative emissions (GtCO2/year). Green dashed lines show the median trajectories for 7 concentration-based groupings of WG3 database scenarios^4^.

In this paper, we explicitly decompose and decouple the CO_2_ emissions contributions of technologies that solely decarbonize the energy system from those that generate negative emissions, and characterize their relative contributions to the magnitude and duration of temperature target overshoot. While the scenarios presented in the WG3 database are obviously not an exhaustive representation of all potential future emissions trajectories, they do encompass a wide range of economically-constrained technological and policy pathways. We therefore use multi-model mean scenarios from the database as guideposts to parameterize the evolution of decarbonization and negative emissions, extending them beyond 2100. We then examine the ranges of scenarios that result in overshoot of 1.5 °C and 2 °C temperature targets. Scenarios (Fig. 1) are indexed by two free parameters: deployment rates for each technology type and medium- to long-term technology deployment targets (see Methods). While agnostic about the particular technologies deployed within either group, the decoupling of these two technology types reveals implicit technology-associated constraints on limiting temperature target overshoot and how those constraints may translate into climate risk assessment.

## Results

**Figure 2:**
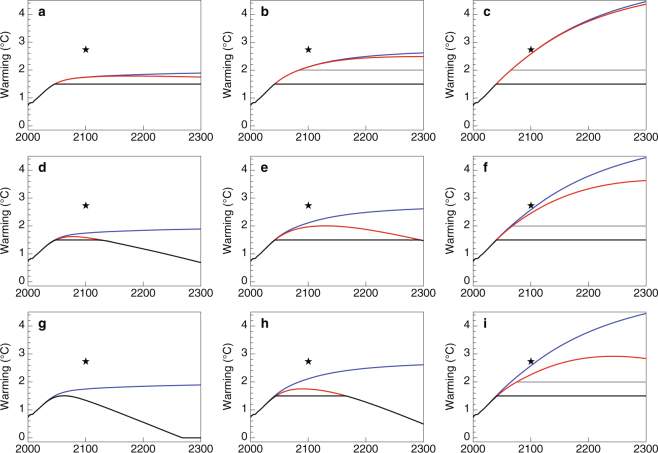
Mean global temperature above preindustrial under a range of decarbonization and negative emissions pathways. Lines show global temperature increases above preindustrial (1861–1880) with decarbonization only (blue), decarbonization and negative emissions (red). Horizontal lines mark 1.5 °C (black) and 2 °C (grey) above preindustrial. Panels show scenarios associated with aggressive (a,d,g), moderate (b,e,h), and weak (c,f,i) decarbonization and weak (**a**–**c**), moderate (**d**–**f**), and aggressive (**g**–**i**) negative emissions deployment. Aggressive and weak scenarios correspond to the end cases in Fig. 1, while the moderate scenario represents the median. Stars indicate the “INDC (conditional)” temperature estimates in 2100 presented in Rogelj *et al*. (2016).

Figure 2 shows the global temperature time series projected under a range of decarbonization pathways only (blue), and combined decarbonization and negative emissions pathways (red) relative to two temperature targets −1.5 °C and 2 °C. Temperatures are projected through 2300 using a simple carbon-cycle-climate model with climate sensitivity to CO_2_ emissions indicative of that simulated in contemporary model intercomparison projects (see Methods). Panels show nine potential scenarios employing aggressive, moderate and weak decarbonization (left to right) and negative emissions (bottom to top) deployments. Characterizations of “weak” and “aggressive” deployments are qualitative, so these designations may be best understood relative to bookend policy benchmarks. First, using a model with median climate response, the combination of the most aggressive decarbonization and most aggressive negative emissions scenarios yields a sole temperature trajectory without overshoot of 1.5 °C. The least aggressive decarbonization scenario with no negative emissions yields a temperature trajectory consistent with Intended Nationally Determined Contributions (INDC) commitments under the Paris Agreement in 2100^12^ (designated by the stars in Fig. 2). In our median case of moderate decarbonization and negative emissions Fig. 2e), global temperature increase is limited to 1.5 °C only after a two century period of overshoot, with the global temperature anomaly peaking at 2 °C degrees early next century. In the least aggressive combination of scenarios, global temperature still hasn’t peaked in 2300. (See Supplementary Figures S1 and S2 for the same figure but with carbon-climate system responses at the high and low end of the IPCC “very likely” range.)

**Figure 3:**
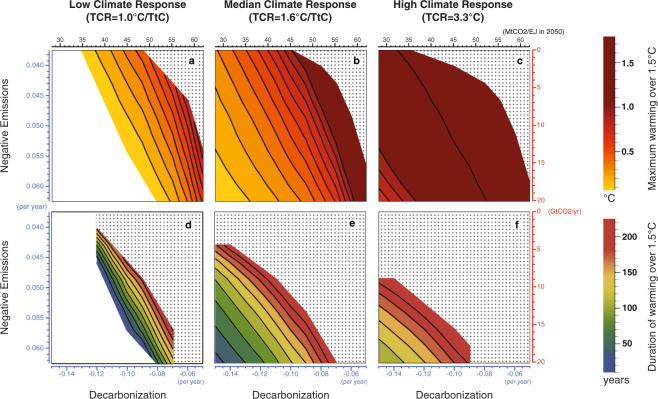
Magnitude and duration of 1.5 °C temperature target overshoot for 5–95% range of climate response. Contours show the interpolated maximum magnitude (in °C) (**a**–**c**) and duration (in years) (**d**–**f**) of the period of overshoot beyond 1.5 °C as a function of decarbonization (indexed by growth rate and a mid-century benchmark, see Methods) and negative emissions implementation (indexed by growth rate and maximum deployment, see Methods) with the highest rates of decarbonization and negative emissions towards the origin. White areas show scenario spaces with no overshoot, and stippled areas scenario spaces where the quantity is still undefined in 2300. Low, median and high climate responses correspond to transient climate responses (TCRs) of 1.0 °C, 1.6 °C and 3.3 °C.

Figure 3 shows the dependence of the magnitude and duration of temperature overshoot on the aggressiveness of decarbonization and negative emissions for a range of climate sensitivities. Whilst decarbonization plays a larger role than negative emissions in limiting the magnitude of overshoot, negative emissions are an essential determinant of its duration. The dynamics underlying this are fairly straightforward; because there is only a lag of about a decade after an emission of carbon dioxide before it has its maximum warming effect^16^, the amount of carbon dioxide released is the primary determinant of maximum warming. At the present day, negative emissions are essentially zero, while positive ones are high, resulting in the net CO_2_ release being mostly driven by the carbon intensity of the energy system in the time period leading up to peak warming. However, because the carbon cycle is slow to remove excess atmospheric CO_2_ naturally, capacity to deliberately remove it becomes a constraining factor in bringing temperature down after a target is surpassed. While in our baseline scenarios the rate and maximum deployment of negative emissions deployment covary, it is total capacity for negative emission that is important for determining overshoot duration, rather than rate of deployment that drives this result (see Supplementary Figure S3). Without sustained negative emissions, even a very small-magnitude overshoot will persist for many centuries.

If Transient Climate Response (TCR), a transient measure of climate sensitivity, turns out to be on the lower end of an assessed 5–95% range^17^ (Fig. 3a and d), a wider range of technology deployments—including those that don’t rely heavily on negative emissions—avoid overshoot altogether. On the other hand, if TCR is on the higher end of the range (Fig. 3c and f), the scenarios that result in overshoot are essentially the same, but its magnitude is much higher.

These quantitative results are sensitive to scenario-driven assumptions. For example, when implemented in combination with aggressive mitigation of non-CO_2_ forcers (as represented by the emissions pathway associated with scenario RCP2.6), a broader range of aggressive decarbonization and carbon removal scenarios avoid overshoot altogether (see Supplementary Fig S4). Likewise, lower or higher energy demand scenarios broaden or restrict pathways to 1.5 °C or 2 °C temperature targets (Supplementary Figs S5–S6). However, the qualitative results are robust to alternative formulations. The magnitude of the overshoot temperature is largely determined by the aggressiveness of decarbonization policy, while the duration is driven by the scale of both decarbonization and long-term negative emissions deployment levels.

## Discussion

In the range of futures explored in the WG3 scenario database, under a mid-range climate response overshoot is a virtual certainty in nearly all pathways to achieving a 1.5 °C temperature target by the end of the 22^nd^ century. For a 2 °C target (Supplementary Figure S7), there are also a limited range of moderate decarbonization conditions that yield a limited period of overshoot. Generally, in this WG3-constrained range of decarbonization and negative emissions scenarios the 2 °C target is either met or exceeded for many centuries. When overshoot does occur, its magnitude is generally less than 1 °C. This suggests that understanding the mechanistic drivers of global temperature-linked climate change impacts (i.e., threshold-based versus cumulative damage) may be particularly important in the context of the post-Paris emphasis on a 1.5 °C target.

The carbon-cycle-climate model (Finite Amplitude Impulse-Response Model, or FAIR)^18^ used to simulate the temperature implications of combined decarbonization and negative emissions futures accounts for the state-dependence of the temperature and concentration response to additional CO_2_ emissions. FAIR successfully emulates the behavior of widely-used MAGICC simple climate model^19^ under the RCP2.6 scenario, which involves net negative emissions at the end of the century (see Millar *et al*. 2017). However, an important caveat to our results is that whilst such simple models may represent the climate system well over the historical period (when emissions have been increasing), caution should be taken in interpreting the projections of a simple model when emissions and radiative forcing are declining^20^. As substantial negative emissions are deployed here, the symmetry between the response for positive and negative CO2 emissions is important. In Figure S8 we show the response of the simple carbon-cycle-climate model to the negative emissions scenarios of Jones *et al*.^21^. Our model performs similarly to the simple model used there and the efficacy of negative emissions displays similar dependencies on the background emissions scenario as seen there. Our model also displays a declining efficacy of negative emissions with additional negative emissions as seen for the UVic Earth System Climate Model^22^. A comprehensive assessment of the dependencies of the climate response on the magnitude of negative emissions and background climate state across Earth System Models would be useful to help calibrate simple climate carbon-cycle models to the range of behavior seen in more physically based models.

The COP-21 climate meeting in Paris acknowledged the potential value in a more aggressive goal of limiting global mean temperature rise to no more than 1.5 °C without tying specific impacts reductions to this goal. An increasing body of climate change impacts research suggests that 2 °C of global warming will still result in significant damage to infrastructure, human livelihoods, and managed and natural ecosystems, perhaps substantially greater than those associated with 1.5 °C warming^23,24^. Some impacts are driven by threshold behavior, while others are the result of long-term cumulative damage. Our analysis shows a wide range of plausible pathways toward 1.5 °C, and the impacts implications of a long, moderate overshoot versus a short, high magnitude one may well be quite different.

Deployments of decarbonizing and negative emissions technologies have different implications for how global temperature change can be limited, and thus, the timing and magnitude of any temperature target overshoot. Avoiding more than 1.5 °C of warming this century appears to be unlikely through decarbonization alone^11^. Yet large uncertainties are associated with the requisite supplemental contributions from negative emissions technologies to meeting that target. While BECCS and other negative emissions technologies are not completely untested, they have not progressed beyond the pilot project stage^25^, whereas decarbonizing technologies are already deployed to produce a substantial fraction of electricity supply – making them more of a known quantity in terms of economics, policy and regulation. Limiting duration of overshoot is going to depend heavily upon the amount of total negative emission capacity that can be deployed in a technically, ecologically and economically sustainable way. If scalable technologies for negative emissions fail to emerge, then there would likely be substantial and long-lasting overshoot of 1.5 °C. (We do not consider here the use of solar geoengineering^26^ to avoid overshoot^27^, as these technologies remain highly uncertain, contested, and require their own impact assessment.)

Limiting warming to 1.5 °C will be a significant challenge. To date, attributable anthropogenic warming has been calculated to be already in excess of 0.9 °C^28^. Committed warming, including future emissions implicitly committed by existing infrastructure, is estimated to be between 1 and 1.5 °C^29^. The analysis above supports the finding that there are substantial, if not insurmountable, hurdles to meeting a 1.5 °C temperature target without overshoot. A broad range of pathways are associated with a long-term 1.5 °C temperature target – many of which include centuries of exceedance absent aggressive deployment of negative emissions technology. While adjustments to decarbonization targets could effectively manage the magnitude of overshoot, once a target is surpassed, negative emissions technology – in particular, its long-term maximum deployment—is equally essential to limiting duration.

At the request of the UNFCCC, IPCC is preparing a report on the science of a 1.5 °C temperature target^30^. As the climate science community proceeds with a new round of research and assessment with this threshold in mind, it will be important to understand the drivers behind various global temperature linked impacts and how these are influenced by both the duration and magnitude of a temporary overshoot of the target. Certain impacts of climate change will depend not just on long-term temperature targets, but also on the temporal pathway to meeting that target. Understanding how assumptions about technology deployments translate into constraints on the magnitude and duration of overshoot will allow policy makers to better link climate policy goals to specific technological needs.

## Methods

Carbon dioxide emissions scenarios were generated using simple parameterized logistic functions benchmarked using the range of policy scenarios represented in the IPCC WG3 scenario database in which atmospheric CO_2_ concentrations reach between 430 and 1000ppm in 2100^3^.

The WG3 database includes some scenarios using negative emissions, specifically BECCS, and, to a certain extent, land-use change approaches such as reforestation. These are the only two sources of negative emissions in the WG3 database. Because negative emissions-associated land use changes and development-associated land use changes are not reported separately in the database, as an accounting matter, land use emissions are only included as negative emissions when those emissions are net negative. (As explained below, net positive land use emissions are indexed in conjunction with decarbonization scenarios.) Negative emissions pathways as represented in the WG3 database are approximated as:

$$\begin{array}{c}{\rm{Negative}}\,{{\rm{CO}}}_{2}\,{\rm{emissions}}={\rm{Primary}}\,{\rm{Energy}}({\rm{BECCS}})\ast 0.075{\rm{GtCO}}2/{\rm{EJ}}\\ \quad +\,{\rm{Min}}[\{{\rm{Land}}\,{\rm{use}}\,{\rm{CO}}2\,{\rm{emissions}},0\}]\end{array}$$

Negative CO_2_ emissions from BECCS is not a reported variable in the database, so a scaling factor from the literature was used to approximate it from the primary energy generated through BECCS^4,31^.

We assume positive CO_2_ emissions in the scenarios are a function of three variables: carbon intensity of energy generation, primary energy demand, and net positive emissions from land use change:

$$\begin{array}{c}{\rm{Positive}}\,{{\rm{CO}}}_{2}{\rm{Emissions}}={\rm{Carbon}}\,{\rm{intensity}}\,{\rm{of}}\,{\rm{energy}}\,{\rm{x}}\,{\rm{Primary}}\,{\rm{Energy}}\\ \quad +\,{\rm{Max}}[\{{\rm{Land}}\,{\rm{useCO}}2\,{\rm{emissions}},0\}]\end{array}$$

Carbon intensity was the proxy used in our analysis for decarbonization and was calculated from WG3 database values for each scenario using the following formula:

$$\begin{array}{c}{\rm{Carbon}}\,{\rm{Intensity}}=({{\rm{CO}}}_{2}{\rm{Emissions}}+{\rm{Primary}}\,{\rm{Energy}}({\rm{BECCS}})\ast 0.075{\rm{GtCO}}2/{\rm{EJ}}\\ \quad \mbox{--}{\rm{CO}}2\,{\rm{Emissions}}\,{\rm{from}}\,{\rm{land}}\,{\rm{use}})/({\rm{Primary}}\,{\rm{Energy}})\end{array}$$

Negative emissions are added back into the global CO_2_ emissions to avoid double-counting. The mean carbon intensity pathways associated with the 7 scenario groupings of CO_2_ concentration in 2100 used to characterize the WG3 database are shown in green in Fig. 1a. For simplicity, scenarios presented in the main text are generated using global energy demand represented by a single fit to the median primary energy scenario (Supplementary Fig S5). (Supplementary Figure S6 shows results with high and low energy demand pathways.)

A general model of the adoption of new technology types shows usage approximating a sigmoidal, or S-shaped curve^32^. The growth rate and saturation are limited by factors such as technological features, costs, or policy and infrastructure constraints. To approximate this sigmoidal adoption model, pathways were approximated using three or four parameter logistic functions in order to extend a corresponding range of emissions and negative emissions scenarios beyond 2100 where the WG3 database scenarios end.

The generalized form of this function (with different free and fixed parameters for carbon intensity, negative emissions and primary energy) is:

$${\rm{F}}({\rm{t}})={\rm{A}}+\frac{{\rm{K}}-{\rm{A}}}{{(1+{{\rm{e}}}^{-B\ast ({\rm{t}}-{t}_{{\rm{ref}}})})}^{1/{\rm{v}}}}$$

The parameters are upper and lower asymptotes, K and A; a maximum growth rate, B; reference time, t_ref_; and asymmetry parameter, v.

Our high and low parameterized scenarios were fit to correspond to high and low scenarios from the WG3 database. As such, the free parameters covary in the scenarios tested in the main text, but we use sensitivity analyses to test the independent impact of each parameter on the impact of negative emissions deployment (see Supplementary Information). For both carbon intensity and negative emissions, the intervening scenarios were spaced evenly in the assumed growth rates (blue axes in Figs 2 and 3). For carbon intensity, scenarios were also spaced evenly in their projected value in 2050 (black axis). For negative emissions, scenarios were spaced evenly in the upper asymptote that represents a maximum annual rate of atmospheric CO_2_ removal (red axis). For negative emissions, scenarios span the WG3 database range and exceed it on the upper end of the range in order to partially account for negative emissions technologies not yet incorporated into integrated assessment models at the time of the database’s publication. However, contemporary estimates of 12–15 GtCO_2_/year^4,6^ upper limits for negative emissions are consistent with the aggressive scenarios. Negative emissions are fixed at zero before 2020 and phased in linearly through 2040 to remove empirically inconsistent low tails before the present-day.

For simplicity, energy demand is assumed to be identical under all scenarios. A fit to the median primary energy pathway was used as the fixed future primary energy trajectory (see Supplementary Figure S5). A linear representation of positive land use emissions is indexed in conjunction with decarbonization scenario. (See Supplementary Figure S9.) In total we generate 11 decarbonization scenarios and 11 negative emissions (including zero), for a total of 121 scenarios, illustrated in Fig. 1.

For all non-CO_2_ forcings, we add the estimated radiative forcing associated with emissions scenario RCP4.5. We test the sensitivity of our results to this assumption by duplicating the analysis with RCP2.6 values for non-CO_2_ forcings in the Supplementary materials.

To associate the emissions pathways associated with temperature outcomes through 2300 we use a one-dimensional global-mean climate response model designed to emulate the behavior of more complex earth system models^18^. A range of climate sensitivities is evaluated by varying the transient climate response (TCR) of the model between 1.0–3.3 °C with an assumed median response of 1.6 °C from an observationally-constrained 5–95% uncertainty range ^17^ (but similar to the 1.0–2.5 °C likely range as assessed by IPCC^33^). Contemporary uncertainty about climate sensitivity is driven, among other reasons, by large uncertainty about the magnitude of negative non-CO_2_ climate forcings (primarily from tropospheric aerosols) that counterbalance positive CO_2_ forcings. To present projections with a range of TCRs, whilst maintaining approximate consistency with the present-day climate state, the effective radiative forcing of anthropogenic aerosols (both past and present) is multiplicatively scaled following the identical protocol of Millar *et al*.^34^ to reflect the anti-correlation between net anthropogenic forcing and climate response required to accurately simulate a 2015 attributable warming of 0.95 °C above pre-industrial^28^. CO_2_ emission scenarios – which were generated relative to the now partially-obsolete WG3 database— are harmonized to model-derived RCP8.5 values in 2015 using scaling factors that decay to zero in 2050^35^ in order to anchor all generated scenarios to a common 2015 initialization point.

Plots in Fig. 3, Supplementary Figures S3–S4, and Supplementary Figures S6–S7 are generated using first-order linear interpolation to generate contours.

### Data Availability

All the emissions and warming scenarios associated with the main text and supplementary sensitivity analyses are available as Supplementary Data online.

## Electronic supplementary material

Supplementary Information

Dataset 1
