# Supplementary material for: Constraints on global temperature target overshoot
Source: Sci Rep. 2017 Nov 7;7:14743. doi: 10.1038/s41598-017-14503-9 (PMC5676680; doi:10.1038/s41598-017-14503-9)
Supplement: Supplementary file 1 — Supplementary Information [file 41598_2017_14503_MOESM1_ESM.pdf]

Supplementary Information: Constraints on global temperature target overshoot

K.L. Ricke*[a,b], R.J. Millar[c], D.G. MacMartin[d]

a. Scripps Institution of Oceanography, University of California, San Diego

b. School of Global Policy and Strategy, University of California San Diego

c. Department of Physics, University of Oxford

d. Mechanical and Aerospace Engineering, Cornell University

*kricke@ucsd.edu

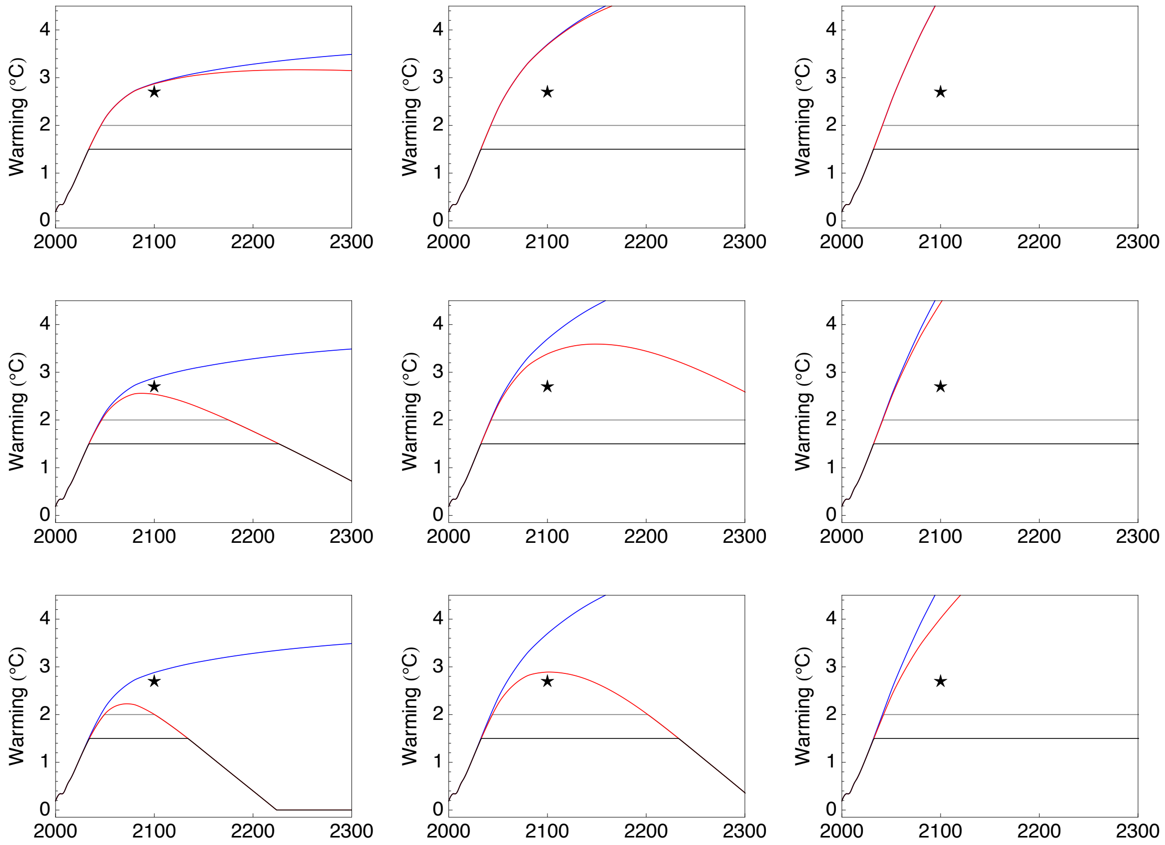

Figure S1. Mean global temperature above preindustrial under a range of decarbonization and negative emissions pathways under high climate sensitivity. As in Figure 2, but for TCR=3.3°C. Lines show global temperature increases above preindustrial with decarbonization (blue), decarbonization and negative emissions (red). Temperature target cut-offs are shown for 1.5 degrees (black) and 2 degrees (grey). Panels show scenarios associated with increasingly aggressive decarbonization (right to left) and negative emissions deployment (top to bottom). Note, stars from Fig.2 remain for comparison but are no longer consistent in terms of climate sensitivity.

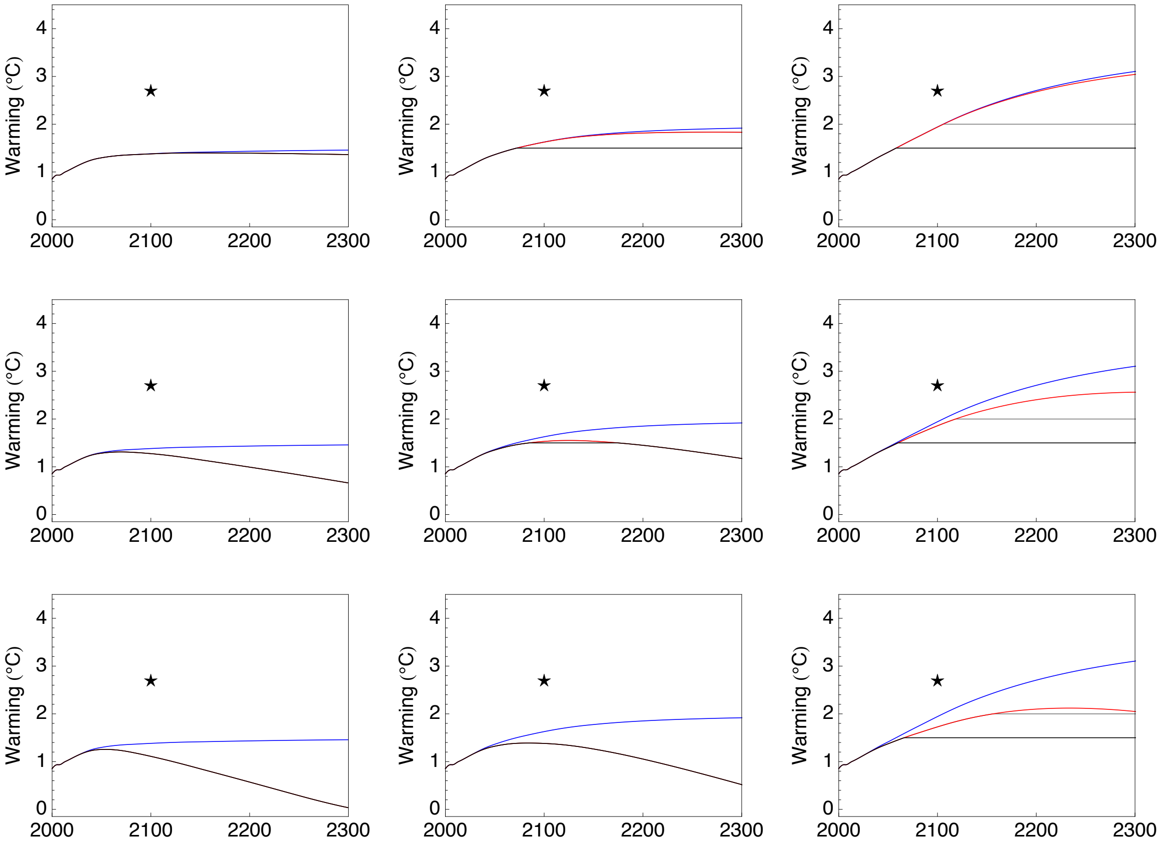

Figure S2. Mean global temperature above preindustrial under a range of decarbonization and negative emissions pathways under low climate sensitivity. As in Figure 2, but for TCR=1.0°C. Lines show global temperature increases above preindustrial with decarbonization (blue), decarbonization and negative emissions (red). Temperature target cut-offs are shown for 1.5 degrees (black) and 2 degrees (grey). Panels show scenarios associated with increasingly aggressive decarbonization (right to left) and negative emissions deployment (top to bottom). Note, stars from Fig.2 remain for comparison but are no longer consistent in terms of climate sensitivity.

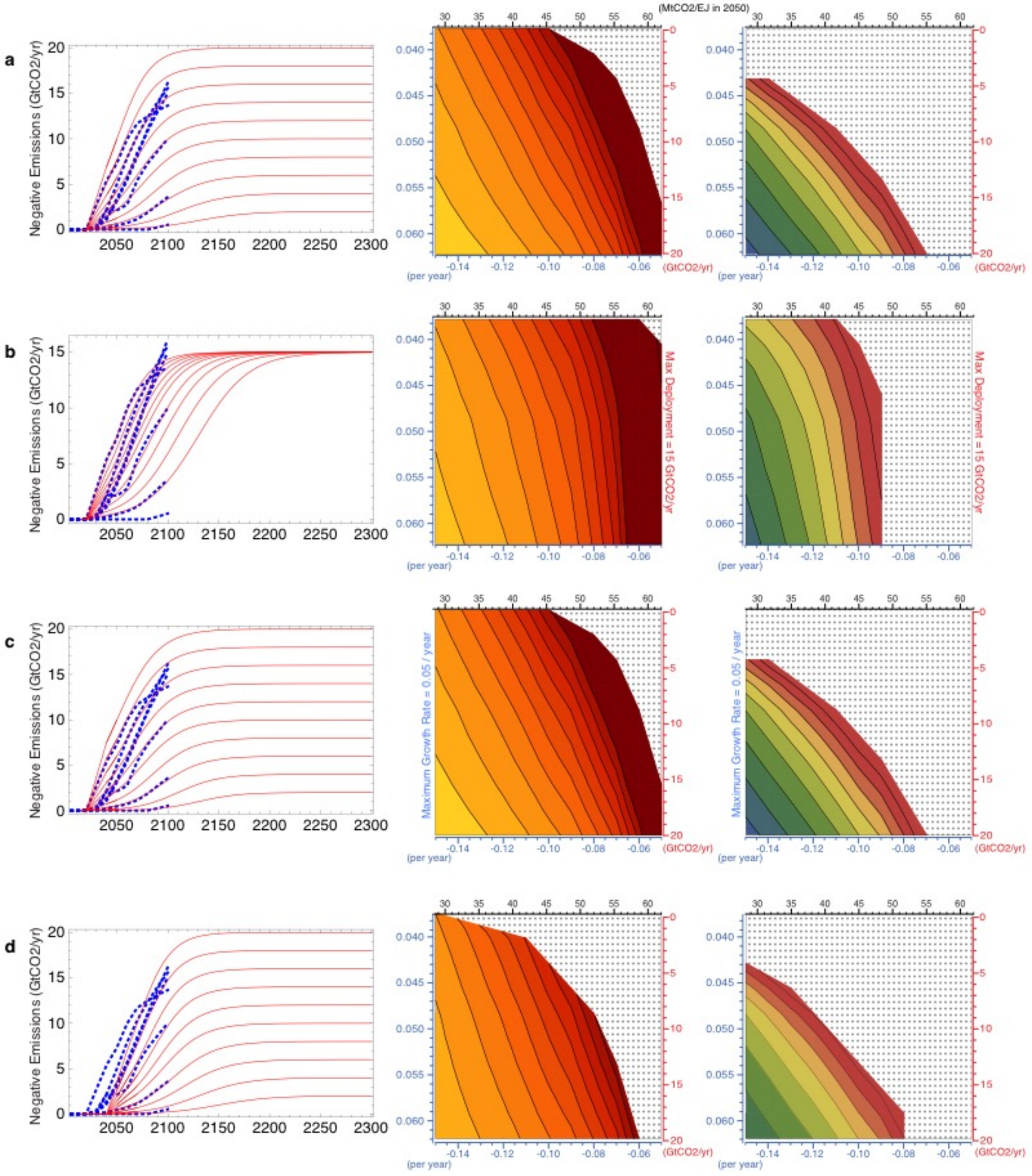

Figure S3. Magnitude and duration of 1.5°C temperature target overshoot for alternative negative emissions scenarios. Negative emissions scenarios, and associated maximum magnitude (in °C) and duration (in years) of overshoot: (a) as in Figure 3, (b) with fixed maximum deployment of 15 GtCO2/yr, (c) with fixed maximum growth rate of 0.05, and (d) as in (a) but with a 20 year lag.

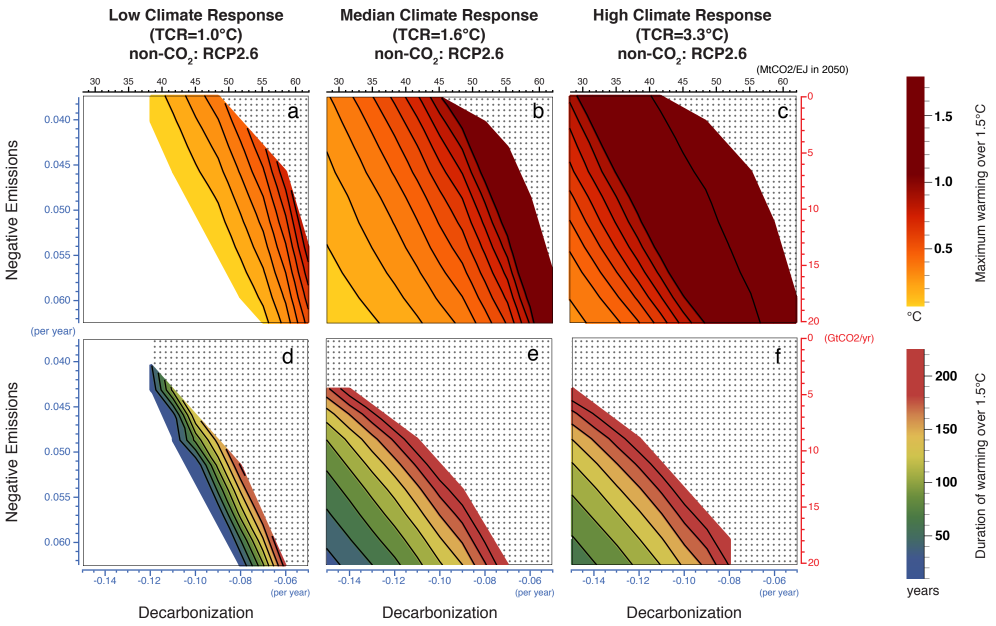

Figure S4. Magnitude and duration of 1.5°C temperature target overshoot for "very likely" range of climate response, using RCP2.6 non-CO_2 forcing. Contours show the maximum magnitude (in °C) (a-c) and duration (in years) (d-f) of the period of overshoot beyond 1.5°C as a function of decarbonization (indexed by growth rate and a mid-century benchmark) and negative emissions implementation (indexed by growth rate and maximum deployment). White areas show scenario spaces with no overshoot, and stippled areas scenario spaces where the quantity is still undefined in 2300. Low, median and high climate responses correspond to TCRs of 1.0°C, 1.6°C and 3.3°C/TtC.

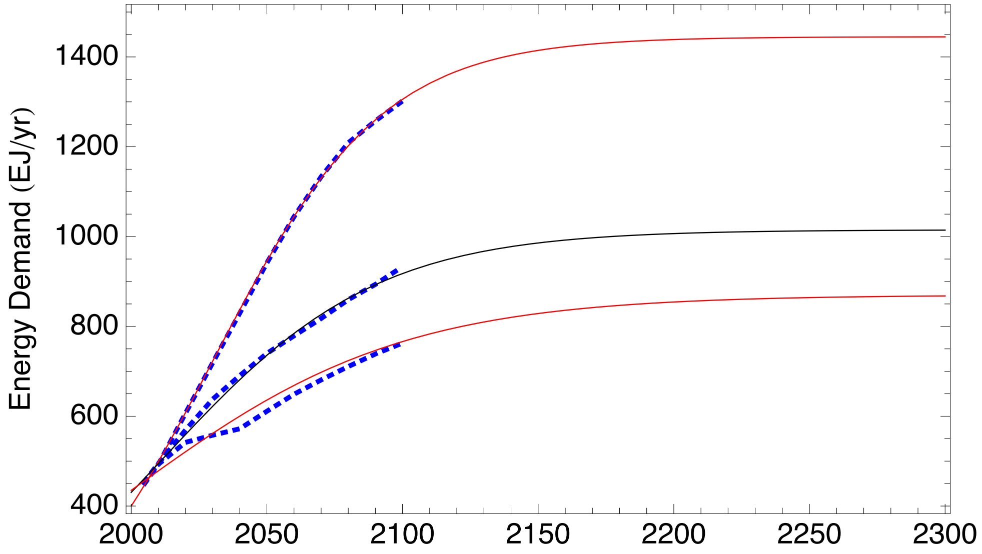

Figure S5. Primary energy demand scenarios. Black line shows the primary energy demand scenario used in the analysis presented in the main text, which is the median for the WG3 database scenarios. Red lines show the high and low energy demand scenarios used to make the projections presented in Figure S6. Blue dashed lines show the median trajectories for high, median and low concentration-based groupings of WG3 database scenarios

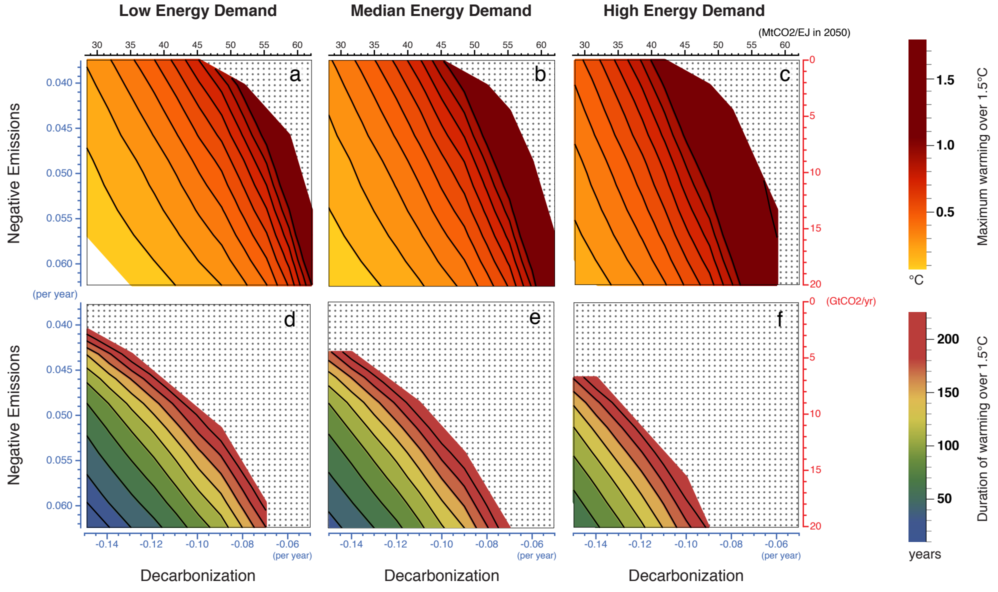

Figure S6. Magnitude and duration of 1.5°C temperature target overshoot for alternative energy demand scenarios. As in Fig. 3 (b) and (e), but with low (a,d) and high (c,f) energy demand scenarios, as illustrated by the red lines in Figure S5.

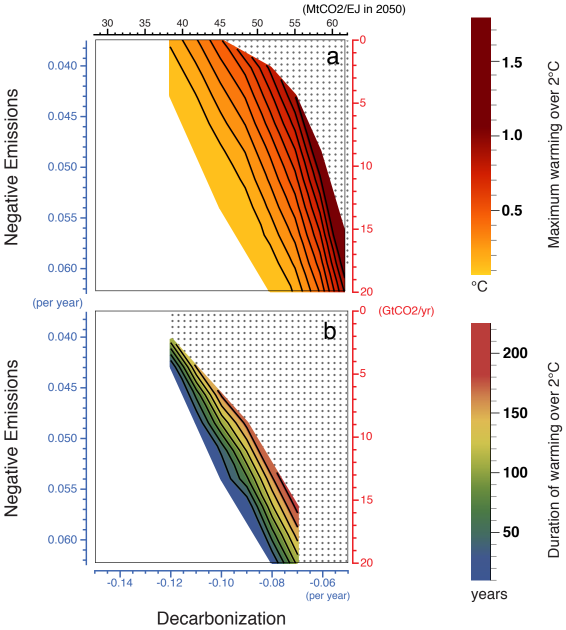

Figure S7. Magnitude and duration of 2°C temperature target overshoot for median climate sensitivity. Contours and colors for (a) and (b) as in Fig. 3 (b) and (e), but for a 2°C temperature target.

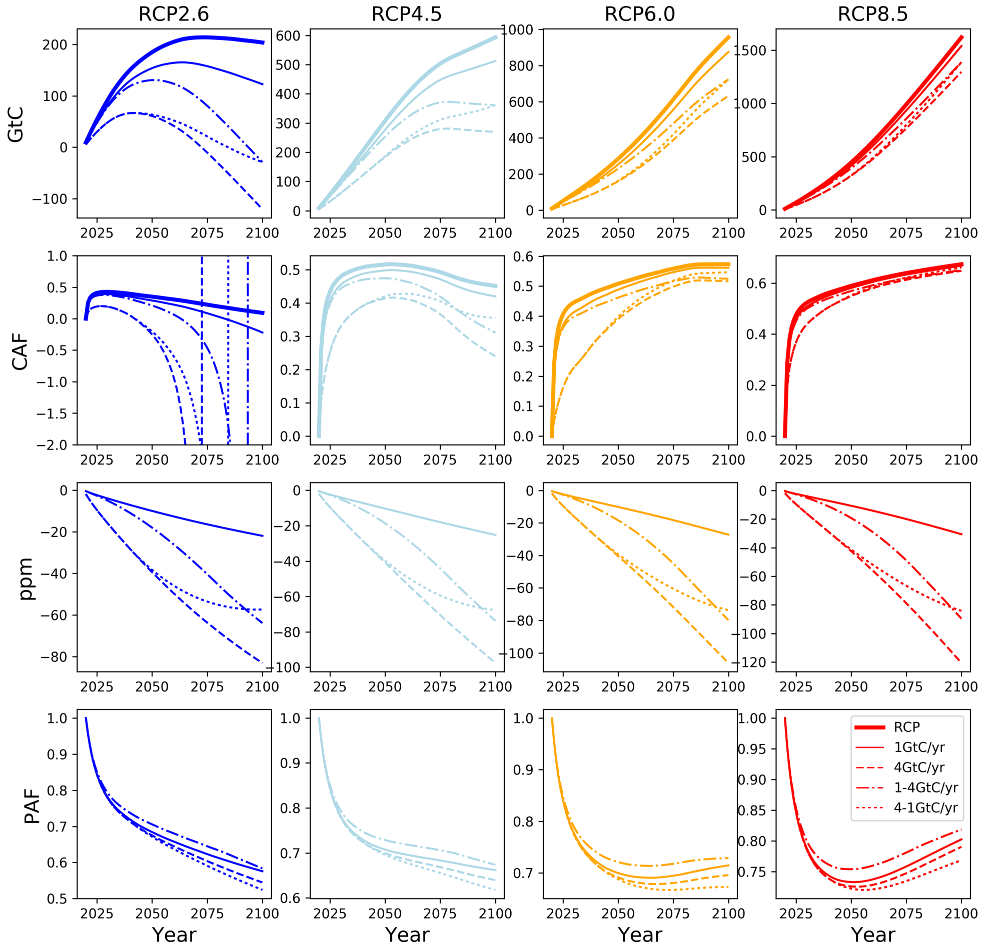

Figure S8: The response of the FAIR model to the negative emissions scenarios of Jones et al 2016. The top row shows cumulative emissions from 2020, the second row the cumulative airborne fraction (CAF - the fraction of cumulative emissions from 2020 remaining in the atmosphere), the third row the change in atmospheric concentration from 2020 and on the bottom row the perturbation airborne fraction of negative emissions (PAF - the fraction of the cumulative negative emissions implementation that remains out of the atmosphere at any point in time). See Jones et al for the scenario descriptions.

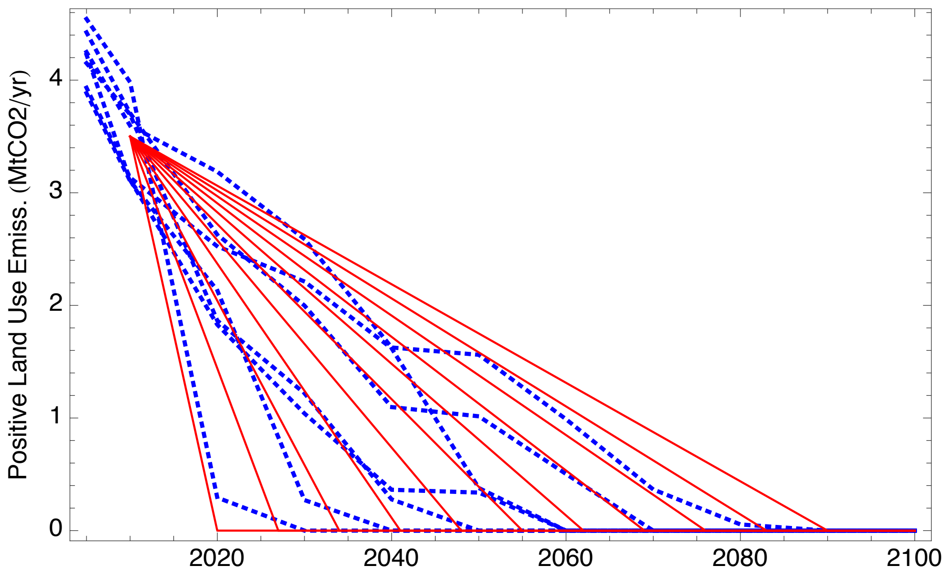

Figure S9. Land-use emissions scenarios simulated. Red lines show the net positive land use emissions scenarios (in $MtCO_2$/year) explored in this analysis, while blue dashed lines show the median trajectories for 7 concentration-based groupings of WG3 database scenarios. Net negative land use emissions are accounted for as a part of negative emissions scenarios (Fig1b).
