# Supplementary material for: Constraints on global temperature target overshoot
Source: Sci Rep. 2017 Nov 7;7:14743. doi: 10.1038/s41598-017-14503-9 (PMC5676680; doi:10.1038/s41598-017-14503-9)
Supplement: Supplementary file 2 — Dataset 1 [file 41598_2017_14503_MOESM2_ESM.zip › SupplementaryData.tar.bz2!/SupplementaryData/readme.rtf]

readme for 
Supplementary Data: “Constraints on global temperature target overshoot”
by K.L. Ricke, R.J. Millar, D.G. MacMartin

The data files in the CO2emissions_scenarios directory contain CO2 emissions time series, in units of GtC, The columns are emissions in the years 2010-2400 and rows are scenarios, grouped by negative emissions, then carbon intensity. 

Files are named as follows:

netco2emissions_010217.csv: baseline scenarios presented in the main text
netco2emissions_010217b.csv: alternative negative emissions scenarios, with maximum deployment fixed at 15 GtCO2/year
netco2emissions_010217c.csv: alternative negative emissions scenarios, with maximum growth rate fixed at 0.05
netco2emissions_010217d.csv: as in netco2emissions_010217.csv but with a 20 year lag
netco2emissions_hied_010217.csv: as in netco2emissions_010217.csv but with high primary energy demand as displayed in FigS5
netco2emissions_loed_010217.csv: as in netco2emissions_010217.csv but with low primary energy demand as displayed in FigS5
netco2emissions_nocdr_010217.csv: as in netco2emissions_010217.csv but with negative emissions fixed at zero
netco2emissions_nocdr_hied_010217.csv: as in netco2emissions_hied_010217.csv but with negative emissions fixed at zero
netco2emissions_nocdr_loed_010217.csv: as in netco2emissions_loed_010217.csv but with negative emissions fixed at zero

The data files in the temperature_projections directory contain temperature projections for these scenarios (harmonised to RCP8.5 through to 2015 with a linear scaling factor, as described in the Methods). The columns are the temperature in the years 1765 through to 2400 (temperatures are relative to 1861-1880) with each row corresponding to the order of scenarios identical to the CO2emissions files. The high TCRE is approximately 2.5K/TtC and the low TCRE 0.8K/TtC. The highrichardson TCRE is 3.3K/TtC and the lowrichardson TCRE 1.0K/TtC. The mid TCRE is approximately 1.6K/TtC. 

Non-CO2 radiative forcing was scaled (within uncertainties) for the different TCRs to correctly capture the present-day state of the climate system, as described in the Methods. 

Files are named as follows:

[emissions scenario]_[non-co2 forcingscenario]_[tcre].csv
